# Influence of the Oil Structuring System on Lipid Hydrolysis and Bioaccessibility of Healthy Fatty Acids and Curcumin

**DOI:** 10.3390/gels10010033

**Published:** 2023-12-30

**Authors:** Susana Cofrades, Joaquín Gómez-Estaca, María Dolores Álvarez, Alba Garcimartín, Adrián Macho-González, Juana Benedí, Tatiana Pintado

**Affiliations:** 1 Institute of Food Science, Technology and Nutrition (ICTAN-CSIC), 28040 Madrid, Spain; jgomez@ictan.csic.es (J.G.-E.); mayoyes@ictan.csic.es (M.D.Á.); 2 Pharmacology, Pharmacognosy and Botany Department, Pharmacy School, Complutense University of Madrid, 28040 Madrid, Spain; a.garcimartin@ucm.es (A.G.); amacho@ucm.es (A.M.-G.); jbenedi@ucm.es (J.B.)

**Keywords:** delivery system structure, olive and chia oils, static in vitro gastrointestinal digestion (GID), fat digestibility, bioactive compound, bioaccessibility, curcumin

## Abstract

Oleogels (OG) and gelled emulsions (GE) were elaborated with a mixture of olive and chia oils (80:20 ratio) without and with the incorporation of the health-related compound curcumin. These were studied to evaluate the influence of the oil structuring system on the lipid hydrolysis and bioaccessibility of three healthy fatty acids (FA) (palmitic, oleic, and α-linolenic acids) and of curcumin, compared to the oil mixture (bulk oil, BO). The oil structuring system influenced the firmness and texture, and the presence of curcumin significantly altered the color parameters. GE showed higher lipid digestibility, with a greater proportion of absorbable fraction (higher content of free FA and monoacylglycerides) than OG, which behaved similarly to BO. The presence of curcumin affected the degree of lipolysis, reducing lipid digestibility in OG and increasing it in GE. As for FA bioaccessibility, although GE presented higher percentages overall, curcumin significantly increased and decreased FA bioaccessibility in OG and GE, respectively. The oil structuring system also influenced the bioaccessibility of curcumin, which was higher in GE. Therefore, when selecting an oil structuring system, their physicochemical properties, the degree of lipid hydrolysis, and the bioaccessibility of both curcumin and the FA studied should all be considered.

## 1. Introduction

Currently, consumer demand is focused on healthier and sustainable products. In this context, the use of vegetable oils as animal fat replacers and the incorporation of bioactive compounds are among the major strategies used to produce different food items (mainly meats and dairy or bakery products). These strategies are based on gelling procedures that offer interesting health benefits [1]. In addition, the technological properties of the final food products can be enhanced by using novel healthy lipid materials as delivery systems. These materials can be obtained through oil structuring techniques based on the gelation process [2]. In this regard, oil-in-water (O/W) emulsion gels (EG) and oleogels (OG) present interesting possibilities. EG may be produced by gelling the continuous phase of a stable, liquid-like emulsion, while OG involve transforming liquid oil into a ‘gel-like’ structure by using an organogelator [2]. From a nutritional perspective, each system is mainly conditioned by its lipid content, which is lower in EG than in OG [3]. In any case, an adequate selection of the lipid source would be desirable for both EG and OG. For instance, the combination of olive and chia oils could be an interesting choice, as oleic and α-linolenic acids would improve the final nutritional quality of the products to which they are added [4]. It is worth noting that, following FAO recommendations, adults should have a minimum total fat intake of 15% of total energy to ensure adequate consumption of essential fatty acids and fat-soluble vitamins. Moreover, the recommended minimum intake for linoleic and α-linolenic acids is 2.5% and 0.5% of total energy, respectively, and between 2.5 and 3.5% for total PUFA [5].

EG and OG systems can function as carriers of healthy fatty acids, depending on the oil or oil mixture used to form their lipid phase. Moreover, they are considered novel vehicles for bioactive compounds, including those with a low aqueous phase. In fact, these systems have shown the ability to solubilize lipophilic compounds within their lipid domains, mainly due to their lipophilic nature [6,7,8,9]. One of these bioactive compounds is curcumin (CU), known for its pharmacological and biological activities, such as anti-inflammatory, anticarcinogenic, and antioxidant properties [10,11,12]. Recent studies have been conducted on developing foods as encapsulation vehicles to effectively delivery CU to specific physiological sites [13]. However, the use of curcumin is conditioned by its hydrophobic nature, low water-solubility, poor chemical stability (especially in alkaline solutions), and relatively high rate of metabolic degradation during physiological transit. These, among other factors, limit its absorption and bioavailability in the human body [14,15]. A promising strategy to improve the bioaccessibility and bioavailability of curcumin after ingestion involves encapsulation using delivery systems, including those based on some types of GE [13] and OG [16,17]. This is because, beyond composition, food structure plays an important role in how it interacts with the components of the gastrointestinal tract (e.g., bodily fluids and receptors), thus influencing the resulting release and uptake of nutrients [18]. In this sense, the lipid digestion and release of bioactive compounds such as curcumin in different emulsions may be potentially influenced by several factors. These include the type of emulsifier/stabilizer used to form emulsions, which affects interfacial phenomena; the oil composition and dispersion conditions of curcumin in the oil phase; the size of the emulsion oil droplets, which affects the surface area available for lipase adsorption; and the consistency of the systems, which influences mass transport [19,20]. In reference to OG, various oleogelators (such as wax, mono- and di-glycerides, ethyl cellulose, and phytosterol) have been studied to assess their impact on lipolysis. These investigations aim to evaluate the relationship between the physical properties of the organogelators and their digestibility [17], as well as their influence on the release of bioactive compounds [7]. In this regard, OG have been widely used as carriers for lipophilic nutrients, including curcumin. Their role is not only to regulate the release rate of the encapsulated substances but also to improve the bioavailability of nutrients [17].

In relation to GE, various authors have demonstrated the potential applications of different oil-in-water emulsion-based approaches, such as conventional emulsions, gel emulsions, nanoemulsions, and Pickering emulsions. These methods have been specifically used to deliver curcumin, improving its stability, bioaccessibility, and bioavailability based on their different structures [13]. Considering the above, understanding the behavior of each system could offer potential solutions to improve consumer health, either by limiting fat digestibility to mitigate obesity or by improving the availability of biocompounds. For this purpose, the objective of the present study was to determine how the structure of the delivery system, oleogels (OG) and gelled emulsions (GE), compared to bulk oil (BO), impacted fat digestibility and the bioaccessibility of three healthy fatty acids (palmitic, oleic, and α-linolenic acids) and curcumin.

## 2. Results and Discussion

### 2.1. Characterization of the Undigested Systems

#### 2.1.1. General Appearance, Color, and Texture

**Figure 1:**
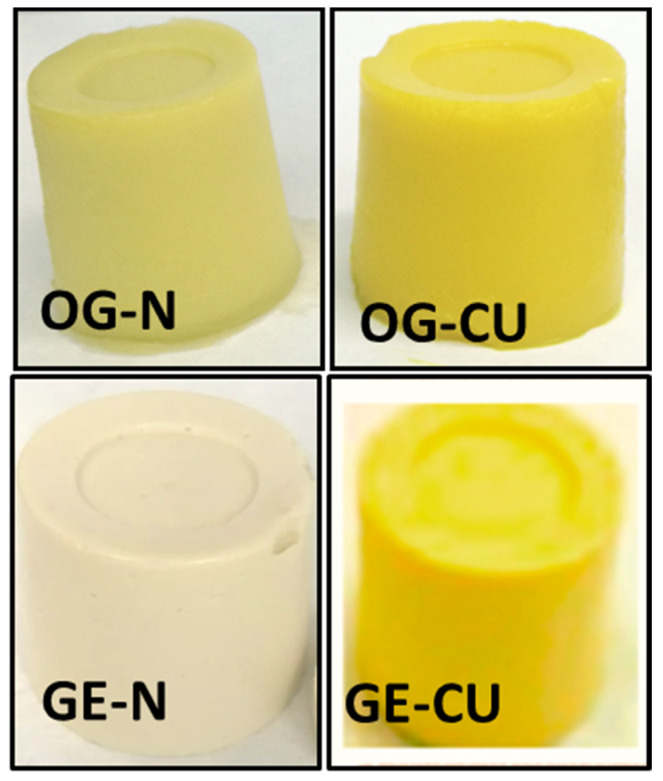
General appearance of the oleogels (OG) and gelled emulsions (GE) in the absence (OG–N and GE–N) and presence of curcumin (OG–CU and GE–CU).

Regardless of the structuring system used to stabilize the combination of olive and chia oils, both methods produced solid-like, smooth, and homogeneous structures (Figure 1). This visual appearance is suitable for their use as substitutes for animal fat [2]. When a vegetable oil (liquid at room temperature) is structured, the color of the oil determines the final color of the structured system. In this sense, all GE and OG were yellow, with intensity variations depending on the amount of oil they contained and the presence/absence of curcumin. Thus, the OG exhibited a more intense color than the GE, as the OG contained a greater amount of the oil mixture (Table 6). Adding curcumin did not affect the solid-like structure (Figure 1) of the system, but it did result in a more pronounced yellow color.

The objective color measurement (Table 1) indicated that lightness (L*) values were higher (*p* < 0.05) in the GE, and, in consequence, these oil-structured systems were lighter. This result is consistent with the visual appearance of the systems (Figure 1). Redness (a*) was higher (*p* < 0.05) in the GE compared to the OG, and, except for GE–CU, all values were below 0. No significant differences were observed in yellowness (b*) between the OG and GE systems. On the other hand, the incorporation of CU led to a significant increase in a* and b*, in line with the yellow color of the CU. These color variations could affect the appearance of the final food products in which curcumin-added delivery systems are used as animal fat replacers [21,22,23].

Texture is a very relevant parameter for the development of animal fat replacers prepared from liquid oils. The OG showed higher penetration force values than the GE, regardless of whether they contained curcumin (Table 1). However, when comparing the hardness of dried meat products formulated with OG or GE as substitutes for pork back fat, those formulated with the OG showed lower values [4]. On the other hand, no significant differences were observed between GE–N and GE–CU, whereas the presence of curcumin increased (*p* < 0.05) the penetration force in the OG. Nonetheless, these differences observed in the OG with and without CU may not necessarily affect the texture of the final product, as observed by Gómez–Estaca et al. [21].

#### 2.1.2. Undigested Systems as Carriers of Fatty Acids with Implications on Human Health

**Figure 2:**
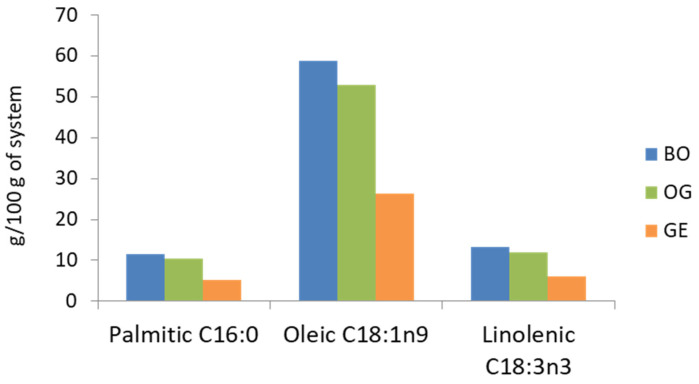
Main fatty acids present in the bulk oils (BO), oleogels (OG), and gelled emulsions (GE) of the undigested systems.

The fatty acid profiles of all undigested systems were similar, as they were elaborated with the same 80:20 mixture of olive and chia oils, and the incorporation of CU, as well as the gelling system, had no effect on this profile. However, since the oil mixture content in the BO was 100%, in the OG was 89 g/100 g, and in the GE was 45 g/100 g (Table 2), the variations observed are attributed to the different amounts of each fatty acid transported by the systems, which depended on their preparation. In this regard, the amount of palmitic, oleic, and α-linolenic acids (the principal fatty acids evaluated) in each system is shown in Figure 2.

Based on the oil mixture used in the oil structuring systems, oleic acid from olive oil was the predominant fatty acid, with contents of 59, 53, and 26.4 g per 100 g in the BO, OG, and GE, respectively. These high oleic contents contribute to healthier nutritional properties compared to products produced with animal fat. In addition, the essential FA α-linolenic acid from chia oil was significantly present, as chia oil is one of the best-known sources of this particular FA. This is interesting in terms of dietary recommendations due to the health benefits it offers [5]. The content of α-linolenic acid in the BO was 9.6 g/100 of sample, while in the OG and GE, the quantity was 8.7 and 4.4 g/100 g of sample, respectively. In this context, using between 20 and 25% of the OG and GE as animal fat substitutes in a meat product would allow reaching approximately 100% and 50% of the recommended daily intake of essential FA α-linolenic acid (2 g/day), respectively. On the other hand, in relation to SFA, palmitic acid was the primary SFA, with its content ranging between 11.5 g/100 g in the BO and 5.2 g/100 g in the GE.

### 2.2. Extent of Lipolysis during Static In Vitro Digestion

Table 2 summarizes the lipid composition after in vitro GID of the two bulk oils (BO–N and BO–CU), the two oleogels (OG–N and OG–CU), and the two gelled emulsions (GE–N and GE–CU). The extent of lipolysis was influenced by the oil structuring system and the addition of CU. To quantify the extent of lipolysis, it was necessary to examine the proportion of unhydrolyzed triacylglycerides (TAG) (Table 2).

Before digestion, the lipid composition was typical of oil, with 99.9% TAG, and there were no significant differences among them. After static in vitro GID, both the delivery systems and the BO suffered changes in their composition. This led to a considerable reduction in the TAG proportion and to an increase in the presence of hydrolytic compounds (diacylglycerides (DAG), monoacylglicerides (MAG), and free fatty acids (FFA)), although to varying degrees depending on the system and the presence or absence of CU. Regarding the oil structuring system and regardless of the presence of CU, the GE showed lower TAG contents and a higher proportion of FFA + MAG (absorbable fraction), and, therefore, higher lipid digestibility than the OG and BO. These results cannot be directly compared to those found in the existing literature due to a lack of studies on similar systems. Nonetheless, Ciuffarin et al. [24] observed an increase in FFA release after in vitro GID when olive oil was structured in a whey protein aerogel, compared to OG elaborated with different organogelators.

The higher degree of lipid digestion of the GE could be attributed to the protein content of the system, as its structure would be partially or completely degraded by the action of proteolytic enzymes. In this case, the emulsion formed during digestion facilitates digestive lipolysis and, thus, lipid bioaccesibility. In contrast, the access of lipases to the BO or OG may be hindered due to their more compact structure. Mulet–Cabero et al. [25] demonstrated the effect of the food matrix structure on nutrient digestion in different dairy products. Therefore, the varying extent of lipolysis observed between the GE and the BO may be related to the smaller droplet size and the larger surface area of lipid droplets exposed to digestive enzymes, as described by different authors [26]. Smaller droplets present a larger surface area, providing a greater number of sites for lipase binding and facilitating lipolysis. These results are in agreement with studies reporting a reasonable correlation between droplet size and rate of lipolysis [27].

In addition, another possible explanation for the higher level of lipolysis observed in the GE vs. the OG could be their different gelation mechanisms, which results in different structures. Therefore, based on the consistency of the GE and OG (Table 1), the softer delivery systems (GE–N and GE–CU) may have undergone degradation much faster than the harder oleogels (OG–N and OG–CU) during in vitro GID. In the case of the OG containing beeswax, FA and TAG assemble into crystals by van der Waals interactions, forming a particle network [28]. The gelation is achieved by crystal cross-linking, effectively trapping the mobile phase [29]. This allows molecules to interact more closely with their neighbor molecules through short-range interactions, resulting in strong and elastic structures [29,30]. In contrast, in this study, the GE were formed by an O/W emulsion stabilized with SPI and gelatin as a gelling agent, which resulted in a weaker structure compared to the OG. This may have led to certain outcomes during gastric digestion. On the one hand, the proteolysis of the interfacial layer may have caused a gradual loss of the superficial charge of the fat droplets and a decrease in the thickness of the interfacial layer, promoting destabilization of the gel structure [31]. On the other hand, as the gel with gelatin transforms into a viscous liquid at 37 °C, this may have weakened the structure of the GE. This would explain the higher degree of lipid digestibility observed in all the GE. In this regard, it has been reported that a slight hydrolysis of gelatin occurs during the first 2 h of static in vitro digestion due to the action of pepsin [32,33].

Curcumin had a different impact on the degree of lipolysis depending on the system to which it was added (Table 2). In the case of the BO, the presence of curcumin had no discernible effect, while in the GE and OG, the effect was significant but varied depending on the structuring system. In the OG, curcumin significantly reduced lipid digestibility. However, in the GE, the presence of this compound notably increased lipid hydrolysis, resulting in values of up to 86% of lipid digestibility with an absorbable fraction of 62% and only 13.7% TAG at the end of the static in vitro GID. This behavior could be associated with the greater degree of lipolysis that the GE may have undergone in comparison to the OG and BO, as mentioned above. Among all the systems examined, the OG loaded with curcumin experienced the least digestion in contrast to BO–CU and GE–CU. This effect could be attributed to the fact that curcumin could have precipitated within the crystalline structure formed by the beeswax and the oil. This may have led to interference with bile function and, consequently, the lipid digestion process. There are a very limited number of studies comparing the effect of different structuring systems (OG vs. GE) with an added bioactive compound, such as curcumin, on the degree of lipolysis during in vitro GID. In this context, Yu et al. [34] developed an organogel and an organogel-based nanoemulsion, both containing curcumin and observed that after in vitro GID, the rate and extent of lipolysis of the nanoemulsion was significantly faster and more complete than that of the organogel. These findings are similar to those of our study, and the authors indicate that the reason could once again be ascribed to the difference in the surface area of the fat particles. Nanoemulsions with smaller droplet sizes have a significantly larger surface area available for lipase-catalyzed lipid hydrolysis, suggesting that the digestion of nanoemulsions is more complete.

### 2.3. Fatty Acids Profile and Bioaccessibility of Evaluated Fatty Acids

Table 3 shows the FFA, grouped by categories (SFA, MUFA, and PUFA), present in Fraction 2 (F2) of the micellar phase of the systems without curcumin (BO–N, OG–N, and GE–N) and with curcumin (BO–CU, OG–CU, GE–CU). First, it is worth noting that, after in vitro GID, the composition of FA corresponds to that observed in the undigested systems, where MUFA predominated. The oil structuring system influenced the composition of these FFA, and the GE contained higher quantities of SFA, MUFA, and PUFA compared to the BO and OG, regardless of the presence of curcumin. No notable differences were found between these two latter systems. As for the effect of curcumin, it was only significant in the case of MUFA in the OG, and PUFA in the GE. In the OG, curcumin could be limiting the digestion of MUFA, whereas in the GE it seems to promote PUFA digestion.

Table 4 displays the bioaccessibility of the main fatty acids with health implications. Both the oil structuring system and the presence of curcumin had an effect on the bioaccessibility of the fatty acids. However, this effect varied depending on the specific fatty acid considered. Regarding the impact of the oil structuring system, both in the absence and presence of curcumin, the GE showed the highest percentages of bioaccessibility for palmitic, oleic, and α-linolenic acids. Once again, this behavior could be linked to the higher degree of lipolysis experienced by the GE compared to the OG and BO. This is because the protein-based gel matrix may undergo partial or more complete digestion when the oil is emulsified. This differs from what is observed when the oil is not emulsified, as in the case of the BO, or when trapped within a crystalline network, such as that formed by the beeswax in the OG [25,32]. In contrast, oleogelation did not affect the bioaccessibility percentages of the fatty acids compared to the BO, as had been observed in the FFA after in vitro digestion (Table 3). In addition, the effect of curcumin incorporation was different in each system (Table 4). In the case of the BO, curcumin exhibited no effect on the bioaccessibility of the three fatty acids. However, in the OG system, the presence of curcumin decreased the bioaccessibility of palmitic, oleic, and α-linolenic acids, whereas in the GE system, it increased the bioaccessibility of both palmitic and α-linolenic acids. Regarding the bioaccessibility of the three selected fatty acids, palmitic acid (SFA) showed the highest values among the three systems studied (BO, OG, and GE). This result could be linked to the fact that during in vitro lipolysis, the short-chain FFA that are released exhibit greater affinity for the aqueous or soluble phase than their long-chain counterparts, which tend to surround fat droplets [32,33]. Consequently, unsaturated fatty acids like oleic and α-linolenic acids, with a higher molecular weight, might be less accessible than SFA like palmitic acid (Table 4).

### 2.4. Bioaccessibility of Curcumin

**Figure 3:**
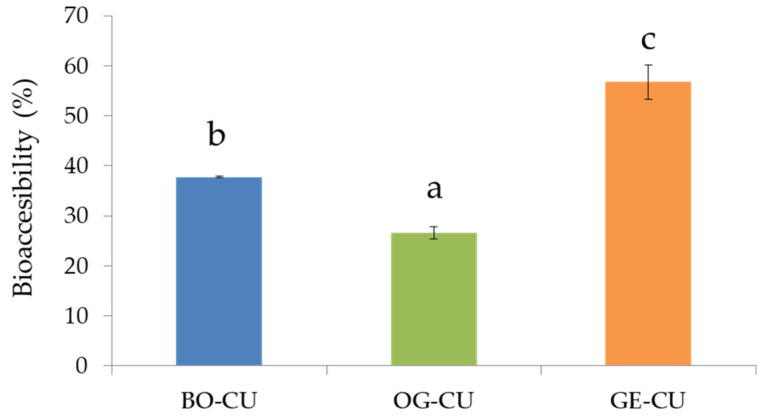
Bioaccessibility (%) of curcumin for the different systems (BO–CU, OG–CU, and GE–CU). Different letters to compare the systems indicate significant differences (*p* < 0.05).

The amount of solubilized curcumin after lipolysis in the three different systems (BO–CU, OG–CU, and GE–CU) was also determined. This study revealed (Figure 3) that the bioaccessibility of curcumin was conditioned by the oil structuring system. The percentage of soluble curcumin with respect to the initial amount in each system was significantly higher in the GE (57%) compared to the BO (38%) and the OG (25%). The high bioaccessibility of curcumin observed in the GE might be linked to the high lipid digestibility observed under intestinal conditions. Although curcumin is a hydrophobic bioactive compound, it is incorporated into mixed micelles to a large extent. This incorporation occurs because mixed micelles are formed by the interactions of the FFA and MAG that are released from the oil droplets [35]. Thus, lipid digestibility could lead to the release of a substantial amount of curcumin. These bioaccessibility values fall within the range of values reported by other authors [13], who found that the bioaccessibility of curcumin incorporated in emulsion-based lipid carriers commonly varies between 20% and 65%, depending on the formulation characteristics (such as the type of lipid employed or the interfacial composition). It is well-established that curcumin must be in its soluble form to enter the micellar phase. Thus, any formulation that prevents the recrystallization of curcumin would be ideal for the delivery of solubilized curcumin. Gelled structures are known to reduce the molecular mobility of solutes dissolved in the continuous phase, thereby minimizing the recrystallization of curcumin within these systems. This, in turn, enhances curcumin delivery, as observed in this study [36]. Similar bioaccessibility values have been observed in nanoemulsions containing 5 mg of curcumin per 100 g of delivery system [37]. Other studies have found no differences in the bioaccessibility of curcumin in emulsion delivery systems containing different levels of proteins or lipids [38].

This wide variety of results found in the literature regarding the bioaccessibility of curcumin and other bioactive compounds incorporated into different delivery systems (GE, OG, O/W emulsions, etc.) could be a consequence of numerous factors. On the one hand, the components that constitute the different carriers, such as proteins, fibers, polysaccharides, oleogelators, etc., and on the other hand, the preparation conditions of these carriers, such as the type and proportion of the oils/fats used and emulsification/gelation processes, among others. In addition, variations in the in vitro GID processes conducted by different authors may have also contributed to the discrepancies observed [39].

### 2.5. Correlations

A Pearson’s correlation test was performed to investigate the correlation between the extent of lipolysis, measured by the disappearance of TAG and the formation of hydrolytic compounds (MAG and FFA), as well as the percentage of bioaccessibility of the main fatty acids (palmitic, oleic, and α-linolenic acids) and that of curcumin (Table 5).

In both structured systems (OG and GE), the percentages of bioaccessibility of palmitic, oleic, and α-linolenic acids were negatively and significantly correlated with TAG and positively and significantly correlated with MAG + FFA (Table 5). In the oil mixture (BO), only the percentage of bioaccessibility of palmitic acid exhibited a similar correlation with TAG and MAG + FFA.

## 3. Conclusions

Overall, the results of the present study confirm the possibility of controlling both lipid digestion and the delivery of fatty acids (palmitic, oleic, and α-linolenic acids) and curcumin using different oil structuring systems. This suggests their applicability as fat replacers, offering not only technological advantages but also the capacity to meet specific nutritional requirements. In this context, the GE presented higher lipolysis and greater bioaccessibility of the three fatty acids studied and curcumin. Hence, a protein-based gelled emulsion system, developed with a healthy oil mixture containing olive and chia oils and with curcumin incorporated, could be a suitable fat replacer. This would be particularly beneficial when developing products to meet recommended intake levels of essential omega-3 fatty acids. Moreover, it would provide the additional advantage of including a compound with antioxidant activity. The results of the present work also indicate the possibility of developing lipid-structured systems with low fatty acid bioaccesibility (by oleogelation) in the development of low-calorie food products. Therefore, the oil-structured systems developed in this study could be used as animal fat replacers and as bioactive compound carriers in the production of healthy meat products designed for specific consumer groups.

## 4. Materials and Methods

### 4.1. System Design and Preparation

Three different systems were prepared and evaluated: oleogels (OG), gelled emulsions (GE), and, as reference, bulk oil (BO) (Table 6). A mixture of extra virgin olive oil (La Española, Aceites del Sur Coosur S.A.; Vilches, Spain) and chia oil (Primaria, Valencia, Spain) in an 80:20 ratio was used to form the lipid phase of the systems. In addition, this mixture was prepared with curcumin (CU) (Sigma-Aldrich (Madrid, Spain). Therefore, the BO sample consisted of a mixture of olive and chia oils without curcumin (BO–N) or with 0.2% of curcumin (BO–CU). Two OG, using beeswax (Guinama, S.L.U., La Pobla de Vallbona, España) as the organogelator were prepared, one without curcumin (OG–N) and the other with 0.2% CU (OG–CU). Moreover, two oil-in-water (O/W) protein-based gel emulsions without (GE–N) and with added curcumin at 0.2% concentration (GE–CU) were also elaborated. Soy protein isolate (SPI) (10%) and gelatin (3%) (type B, 200–220 bloom) from Manuel Riesgo, S.A. (Madrid, Spain), were used as the emulsifying protein and as the gelling agent, respectively, in the elaboration of the GE.

Firstly, the mixture of oils used as the reference sample (BO–N) and to elaborate the structured systems was prepared. Olive and chia oils were mixed in an 80:20 ratio, respectively, by stirring for 10 min at room temperature and in darkness. To formulate BO–CU, curcumin (0.2%) was added to the oil mixture, and to facilitate its solubilization, the mixture was sonicated using an ultrasound probe (Sonicator Ultrasonic processor Part No. Q700; Q-sonica, Newtown, CT, USA) for at least 5 min. The samples (BO–N and BO–CU) were refrigerated (2 ± 2 °C) in darkness until use (less than two days).

The OG formulated without and with curcumin (OG–N and OG–CU, respectively) were elaborated as follows: the respective oil mixtures, without (BO–N) and with CU (BO–CU), as prepared above, were combined with beeswax and mixed using a homogenizer (ThermomixTM 31, Vorwerk, Wuppertal, Germany) under constant stirring at 65 °C until complete melting and mixing. Then, the mixture was transferred into plastic containers (50 mL), allowed to stand for 30 min at room temperature in darkness, and subsequently stored under chilled conditions (2 ± 2 °C) until use. This process allowed complete gelation, resulting in samples OG–N and OG–CU.

The O/W protein-based emulsion gels or GE, without and with curcumin (GE–N and GE–CU, respectively), were elaborated as described below. SPI and pork gelatin, the latter previously dissolved in 1/3 of total water by heating, were used to prepare the GE. The ingredients were mixed at room temperature, using the same homogenizer mentioned earlier, with the gradual addition of the corresponding oil mixture, BO–N for the GE–N sample and BO–CU for the GE–CU sample. Then, the samples were placed into plastic containers (50 mL) and refrigerated (2 ± 2 °C) until use.

### 4.2. Characterization of the Undigested Systems

Solid-like oil-structured systems, which have been widely used as animal fat replacers in the development of different food matrices, are selected based on their composition, technological properties, and sensorial attributes, among other characteristics. In this regard, it is interesting to explore the possibilities that the OG and GE would offer when used as animal fat replacers and bioactive compound carriers.

#### 4.2.1. Color Parameters

The color was measured on oil-structured systems placed on a glass plate using a Konica Minolta CM-3500 D spectrophotometer (Konica Minolta Business Technologies, Tokyo, Japan) set to D65 illuminant/10° observer. The CIELAB color space was used to obtain the color coordinates L* [black (0) to white (100)], a* [green (–) to red (+)], and b* [blue (–) to yellow (+)]. Ten measurements were obtained for each sample.

#### 4.2.2. Texture Analysis

Penetration tests were performed using a TA-XT plus Texture Analyzer (Texture Technologies Corp., Scarsdale, NY, USA) to evaluate the penetration force (N), which represents the maximum force in the force–time curve. The analyses were carried out with a 5 kg load cell and a 4 mm diameter cylindrical stainless steel plunger at a velocity of 0.8 mm/s and up to a depth of 10 mm. Six measurements were obtained for each sample.

### 4.3. Simulated Static In Vitro Gastrointestinal Digestion (GID) of the Systems

BO–N/BO–CU, OG–N/OG–CU, and GE–N/GE–CU were subjected to a simulated in vitro GID following the method described by Laparra et al. [40] with slight modifications. Pepsin, pancreatin, and bile extract, all of porcine origin, were obtained from Sigma Aldrich (Madrid, Spain). To ensure that all samples had the same lipid content, different weights were used: 5 g of BO/BO–CU, 5.5 g of OG/OG–CU, and 11.1 g of GE/GE–CU. Initially, each sample was dissolved in 80 mL of distilled water at 40 °C, and the pH was adjusted to 2 with HCl 6 N. Then, freshly prepared porcine pepsin was added to achieve 2000 U mL^−1^, and the necessary amount of distilled water was incorporated to obtain 100 g of mixture. The samples were incubated (37 °C, 2 h) in a shaking incubator (311DS Labnet, Edison, NJ, USA) set at 120 rpm. Subsequently, the pH was adjusted to 6.5 with NaHCO_3_ 1M. The pancreatin–bile extract, composed of 240 mg of pancreatin (to achieve a lipase activity of 100 U mL^−1^), 550 mg of bile extract, and 100 mg of NaHCO_3_ 1 M, was added to the mixture, and then incubated again at 37 °C for 2 h in the shaking incubator (120 rpm). After that, the reaction was stopped by placing the flasks in an ice bath. The final pH of the solution was adjusted to 7.2 with 0.5 M NaOH. Simulated in vitro GID was conducted six times for each sample: three for determining the extent of lipolysis, and three for evaluating the bioaccessibility of CU and specific fatty acids with significant implications for human health (palmitic acid (PA), oleic acid (OA) and α-linolenic acid (ALA)).

#### 4.3.1. Extent of Lipolysis during In Vitro GID

Once the in vitro GID was completed, fat was extracted from each set of triplicates, as described by Cofrades et al. [27]. Briefly, the systems were mixed with chloroform/methanol (1:1, *v*/*v*) and then washed again with chloroform. The organic phase was purified using a mixture of chloroform/methanol/0.58% NaCl (*v*/*v* 3/48/47), followed by dehydration through filtration using anhydrous sodium sulfate. The solvent was then evaporated to dryness in a water bath at 40–50 °C under a nitrogen atmosphere. High-performance size exclusion chromatography (HPSEC) was conducted to determine the composition and content of TAG, DAG, MAG, and FFA in the fat extracted from the digested samples, as described by Dobarganes et al. [41]. TAG, DAG, MAG, and FFA were quantified as g/100 g of the system. The three samples from each group were measured in duplicate.

The digestibility of the samples was calculated according to the following formula:

$$Digestibility\;\left(\%\right)=\frac{\left({TAG}_{initial}-{TAG}_{t}\right)}{{TAG}_{initial}}\;\times \;100$$

Here, *TAG_initial_* represents the proportion of *TAG* in the oil mixture present in all the undigested systems, and *TAG_t_* is the proportion of *TAG* in the fat of the digestion product at the end of the in vitro GID of each system.

#### 4.3.2. Fatty Acids Profile and Bioaccessibility of the Evaluated Fatty Acids

The final digest solutions of the other three systems were centrifuged (Beckman Avanti tm J-25, Beckman Instruments Inc., Palo Alto, CA, USA) at 15,000 rpm for 30 min at 20 °C. Then, the micellar fraction (the bioaccessible fraction) was collected to determine the bioaccessibility of the evaluated fatty acids (palmitic, oleic, and α-linolenic acids) and curcumin.

From the fat extracted from the micellar fraction of the digested systems, lipid fractionation was performed using SPE columns (Bond Elut NH2 200 mg Agilent Ref 12102089) to isolate Fraction 2, which contained the FFA. The procedure was as follows: approximately 30 mg of the sample was placed in a glass tube, and 0.5 mL of a multipatterned solution (0.1 mg of each pattern) and 3.5 mL of hexane were added. The SPE fractionation was conducted by sequentially eluting 500 μL of the above solution under positive pressure with a syringe. This process included a first phase of chloroform–isopropanol (neutral lipid fraction), a second phase of diethyl ether with 2% acetic acid (FFA fraction), and a third phase of methanol with 2% HCL (phospholipid fraction). The multipattern solution used included the following patterns: triglyceride C13:0 Ref T3882 Sigma Lote SLBS2017V (Purity C13:0 86.5%) for Fraction 1 and total fractions, free fatty acid C19:0 Ref N5252 Sigma Lote 001452535 (Purity C19:0 100%) for Fraction 2, and phospholipid C12:0 Ref P1263 SIGMA Lote SLBK8612V (Purity C12:0 63%) for Fraction 3. The fraction containing FFA was dried under a stream of nitrogen, and once desiccated, it was derivatized to obtain methylated fatty acids (FAME), which were determined by gas chromatography, as described in Section 2.3. Results were grouped into the different fatty acid categories: saturated fatty acids (SFA), monounsaturated fatty acids (MUFA), and polyunsaturated fatty acids (PUFA).

Moreover, the bioaccessibility (%) of palmitic, oleic, and α-linolenic acids, which are the main fatty acids with beneficial health effects, was calculated by dividing the amount of each fatty acid solubilized in the micellar phase by the amount of fatty acid present into the system before the in vitro GID multiplied by100. The analyses to evaluate the fatty acids from undigested samples were carried out using gas chromatography on an Agilent gas chromatograph (Model 7820A, California, CA, USA). Fatty acids were identified by comparing retention times with a fatty acid standard (Supelco 37 FAME Mix 47885-U, Bellefonte, PA, USA). For quantification, an internal standard, C13:0, was added to the sample in its non-methylated state before methylation.

#### 4.3.3. Bioaccessibility of Curcumin

The curcumin present in the undigested systems and in the bioaccessible fraction of the digested systems (BO–CU, OG–CU, and GE–CU) was calculated as described by Gómez-Estaca et al. [42], with some modifications. For that, aliquots of undigested samples were homogenized with ethanol (final ethanol concentration of 80% (*w*/*w*)) using a T-25 Ultra-Turrax blender (IKA-Werke GmbH and Co. KG, Staufen, Germany) and shaken for 2 h at 120 rpm. Then, homogenates were centrifuged (5000 rpm/15 min/20 °C), and curcumin content was determined in the supernatants using UV-Vis spectrophotometry at 428 nm by interpolating in a standard calibration curve of curcumin in 80% ethanol (*w*/*w*) prepared in advance. For curcumin analysis in the bioaccessible fractions, aliquots of the micellar fractions were mixed with ethanol (final ethanol concentration of 80% (*w*/*w*)), shaken for 2h at 120 rpm, and then centrifuged (5000 rpm/15 min/20 °C). Curcumin content was determined using UV-Vis spectroscopy as described before. The percentage of the in vitro bioaccessibility of Cu was determined by dividing the amount of Cu solubilized in the micellar fraction by the amount of Cu present in the system before the in vitro GID multiplied by 100.

### 4.4. Statistical Analysis

Means and standard deviations of data were obtained for all analyses performed. A two-way analysis of variance (ANOVA) was performed to evaluate the statistical significance of the effect of the oil structuring system and the presence or absence of curcumin. A one-way ANOVA, as a function of the oil structured system in the bioaccessibility analysis of CU, was also performed using the SPSS general linear model (GLM) procedure. Tukey’s HSD (honest significant difference) test, with a 95% confidence interval (*p* < 0.05), was used for comparisons of mean values among the oil-structured strategies and the presence of the bioactive component curcumin.

Pearson’s correlations between the extent of lipolysis (TAG and MAG + FFA contents after in vitro GID) and the bioaccessibility of the main fatty acids (palmitic, oleic, and α-linolenic acids) and CU were separately established for each system studied. Analyses were conducted using the IBM SPSS software for Windows, Version 29.0 (IBM Corp., Armonk, NY, USA).

## Figures and Tables

**Table 1:** Instrumental color parameters ((L*) lightness, (a*) redness, and (b*) yellowness) and penetration force values of the oil structuring systems (OG and GE).

	Systems	Without CU	With CU
L*	OG	57.08 ± 0.68 ^a1^	55.23 ± 2.54 ^a1^
	GE	80.02 ± 2.16 ^b2^	72.14 ± 2.54 ^b1^
a*	OG	−3.19 ± 0.07 ^a1^	−2.56 ± 0.27 ^a2^
	GE	−0.20 ± 0.14 ^b1^	1.45 ± 0.76 ^b2^
b*	OG	17.19 ± 0.51 ^a1^	30.45 ± 2.88 ^a2^
	GE	16.06 ± 1.18 ^a1^	53.36 ± 1.20 ^b2^
Penetration Force (N)	OG	0.96 ± 0.01^b1^	1.10 ± 0.02 ^b2^
	GE	0.31 ± 0.05 ^a1^	0.27 ± 0.02 ^a1^

Means ± standard deviation. Different letters to compare the systems and different numbers to evaluate the effect of curcumin in each system indicate significant differences (*p* < 0.05).

**Table 2:** Fat composition and lipid digestibility of the bulk oils, oleogels, and gelled emulsions after in vitro GID.

	Systems	Without CU	With CU
TAG (g/100 g)	BO	51.05 ± 0.52 ^b1^	53.24 ± 3.68 ^b1^
	OG	48.79 ± 5.63 ^b1^	72.56 ± 4.23 ^c2^
	GE	26.42 ± 0.32 ^a2^	13.71 ± 1.63 ^a1^
DAG (g/100 g)	BO	22.32 ± 0.10 ^a1^	21.47 ± 0.88 ^b1^
	OG	21.69 ± 2.96 ^a2^	12.09 ± 0.56 ^b1^
	GE	30.02 ± 1.38 ^b2^	23.98 ± 0.49 ^b1^
MAG (g/100 g)	BO	4.32 ± 0.26 ^a1^	4.31 ± 0.51 ^a1^
	OG	5.01 ± 0.01 ^a2^	3.23 ± 0.69 ^a1^
	GE	18.15 ± 0.40 ^b2^	12.44 ± 0.67 ^b1^
FFA (g/100 g)	BO	22.33 ± 0.35 ^a1^	20.98 ± 2.31 ^b1^
	OG	24.52 ± 2.66 ^a2^	13.13 ± 2.98 ^a1^
	GE	25.42 ± 1.46 ^a1^	49.88 ± 0.48 ^c2^
FFA + MAG (g/100 g)	BO	26.65 ± 0.62 ^a1^	25.29 ± 2.81 ^b1^
	OG	29.53 ± 2.67 ^a2^	15.36 ± 3.67 ^a1^
	GE	43.57 ± 1.06 ^b1^	62.32 ± 1.15 ^c2^
Lipid Digestibility (%)	BO	48.90 ± 0.52 ^a1^	46.70 ± 3.68 ^b1^
	OG	51.16 ± 5.63 ^a2^	32.37 ± 4.23 ^a1^
	GE	73.55 ± 0.32 ^b1^	86.27 ± 1.63 ^c2^

Samples with no curcumin are BO–N, OG–N, and EG–N (bulk oil, oleogel, and emulsion gel, respectively), and samples with curcumin are BO–CU, OG–CU, and EG–CU (bulk oil, oleogel, and emulsion gel, respectively). Means ± standard deviation. Different letters to compare the systems and different numbers to evaluate the effect of curcumin indicate significant differences (*p*< 0.05).

**Table 3:** Free fatty acids grouped by categories present in fraction 2 from the micellar phase of the different systems.

	Systems	Without CU	With CU
SFA (g/100 g)	BO	6.58 ± 1.54 ^a1^	6.24 ± 0.32 ^a1^
	OG	6.55 ± 0.58 ^a1^	4.10 ± 1.35 ^a1^
	GE	11.05 ± 0.42 ^b1^	16.59 ± 0.66 ^b1^
MUFA (g/100 g)	BO	17.36 ± 0.54 ^a1^	11.54 ± 1.32 ^a1^
	OG	18.67 ± 2.36 ^a2^	7.83 ± 2.70 ^a1^
	GE	29.49 ± 2.64 ^b1^	33.94 ± 0.44 ^b1^
PUFA (g/100 g)	BO	6.01 ± 1.11 ^ab1^	4.57 ± 0.48 ^a1^
	OG	5.37 ± 0.62 ^a1^	3.16 ± 1.09 ^a1^
	GE	8.33 ± 0.86 ^b1^	12.84 ± 0.19 ^b2^

Samples with no curcumin are BO–N, OG–N, and EG–N (bulk oil, oleogel, and emulsion gel, respectively), and samples with curcumin are BO–CU, OG–CU, and EG–CU (bulk oil, oleogel, and emulsion gel, respectively) SFA, saturated fatty acids; MUFA, monounsaturated fatty acids; PUFA, polyunsaturated fatty acids. Means ± standard deviation. Different letters to compare the systems and different numbers to evaluate the effect of curcumin indicate significant differences (*p* < 0.05).

**Table 4:** Fatty acid bioaccessibility (%) as a function of the oil structuring system and the presence of curcumin.

	Systems	Without CU	With CU
PA (C16:0)	BO	32.94 ± 4.22 ^a1^	35.19 ± 4.40 ^a1^
	OG	29.03 ± 6.48 ^a2^	19.93 ± 6.86 ^a1^
	GE	49.11 ± 1.03 ^b1^	77.18 ± 2.51 ^b2^
OA (C18:1n9c)	BO	23.51 ± 2.98 ^a1^	16.49 ± 2.34 ^a1^
	OG	21.91 ± 5.18 ^a2^	9.60 ± 3.31 ^a1^
	GE	34.24 ± 1.47 ^b1^	38.96 ± 2.10 ^b1^
ALA (C18:3n3)	BO	20.99 ± 4.44 ^a1^	18.23 ± 2.45 ^a1^
	OG	19.74 ± 4.61 ^a2^	10.46 ± 3.76 ^a1^
	GE	30.61 ± 1.77 ^b1^	41.00 ± 2.39 ^b2^

Samples with no curcumin are BO–N, OG–N, and EG–N (bulk oil, oleogel, and emulsion gel, respectively), and samples with curcumin are BO–CU, OG–CU, and EG–CU (bulk oil, oleogel, and emulsion gel, respectively). PA: palmitic acid; OA: oleic acid; ALA: linolenic acid. Means ± standard deviation. Different letters to compare the systems and different numbers to evaluate the effect of curcumin indicate significant differences (*p* < 0.05).

**Table 5:** Pearson’s correlations between lipolysis products and bioaccessibility (Bio) of the selected FA established in the different systems studied separately.

	TAG	MAG + FFA	Palmitic Bio	Oleic Bio	α-Linolenic Bio	System
TAG	1	−0.993 **	−0.837 *	−0.740	−0.692	BO-N/BO-CU
		−0.997 **	−0.946 **	−0.941 **	−0.942 **	OG-N/OG-CU
		−0.996 **	−0.996 **	−0.956 **	−0.970 **	GE-N/GE-CU
MAG + FFA		1	0.829 *	0.657	0.602	BO-N/BO-CU
			0.960 **	0.953 **	0.953 **	OG-N/OG-CU
			0.999 **	0.975 **	0.986 **	GE-N/GE-CU
Palmitic Bio			1	0.727	0.539	BO-N/BO-CU
				0.998 **	0.997 **	OG-N/OG-CU
				0.966 **	0.979 **	GE-N/GE-CU
Oleic Bio				1	0.959 **	BO-N/BO-CU
					1.000 **	OG-N/OG-CU
					0.998 **	GE-N/GE-CU
α-linolenic Bio					1	BO-N/BO-CU
						OG-N/OG-CU
						GE-N/GE-CU
Curcumin Bio						BO-N/BO-CU
						OG-N/OG-CU
						GE-N/GE-CU

Samples with no curcumin are BO–N, OG–N, and EG–N (bulk oil, oleogel, and emulsion gel, respectively), and samples with curcumin are BO–CU, OG–CU, and EG–CU (bulk oil, oleogel, and emulsion gel, respectively). ** Significant correlation at 0.01 level; * Significant correlation at 0.05 level.

**Table 6:** Ingredients (g/100 g) used in the formulation of the different systems.

Systems	Samples	Oil Mixture	CU	Beeswax	SPI	Gelatin	Water
BO	Without CU (BO–N)	100.0	-	-	-	-	-
	With CU (BO–CU)	99.8	0.2	-	-	-	-
OG	Without CU (OG–N)	89.0	-	11.0	-	-	-
	With CU (OG–CU)	88.82	0.178	11.0	-	-	-
GE	Without CU (GE–N)	45.0	-	-	10.0	3.0	42.0
	With CU (GE–CU)	44.91	0.09	-	10.0	3.0	42.0

BO: bulk oil; OG: oleogels; GE: gelled emulsions; CU: curcumin; SPI: soy protein isolate.

## Data Availability

The data that supports the findings of the current study are listed within the article.
